# Supplementary material for: ProteinShader: illustrative rendering of macromolecules
Source: BMC Struct Biol. 2009 Mar 30;9:19. doi: 10.1186/1472-6807-9-19 (PMC2672931; doi:10.1186/1472-6807-9-19)
Supplement: Additional file 1 — ProteinShader program without source code. This compressed file contains the complete ProteinShader program including associated libraries, but no source code. A README.txt file gives an overview of the ProteinShader distribution, and the index.html file in the help subdirectory has directions on getting started with the program as well as a set of tutorials. [file 1472-6807-9-19-S1.zip › ProteinShader-beta-0_9_4-binary/help/api/org/proteinshader/graphics/typography/TextLabelFactory.html]

TextLabelFactory (ProteinShader API)


|  |  |  |
| --- | --- | --- |
| **Overview** \| **Package** \| **Class** \| **Use** \| **Tree** \| **Deprecated** \| **Index** \| **Help** | |  |
| **PREV CLASS**   **NEXT CLASS** | **FRAMES**    **NO FRAMES**     **All Classes** |  |
| SUMMARY: NESTED \| FIELD \| CONSTR \| METHOD | DETAIL: FIELD \| CONSTR \| METHOD |  |


---


## org.proteinshader.graphics.typography Class TextLabelFactory

```
java.lang.Object
  org.proteinshader.graphics.typography.TextLabelFactory
```

---

``` public class TextLabelFactory extends Object ```

Uses a GlyphSet to create short text labels to place on segments of
a tube or ribbon. The labels will be amino acid abbreviations and
sequence numbers (e.g. 'A 123' for alanine as the 123 amino acid in
a chain).

---

**Constructor Summary**

`TextLabelFactory()`
  
          Constructs a TextLabelFactory.


| **Method Summary** | |
| --- | --- |
| `TextLabel` | `createLabel(String text, int width, int height)`             Uses Glyph objects to create a TextLabel with a Buffer containing an image based on the text String given as an argument. |
| `void` | `readGlyphSet()`             Obtains a set of glyphs by using the default '.conf' configuration file in the fonts directory. |
| `void` | `readGlyphSet(String config)`             Reads the config file given as an argument and use the info to create a GlyphSet. |
| `void` | `setDebug(boolean debug)`             Setting debug to true will turn on debugging (which prints data to standard out) for the GlyphSetFactory. |

**Methods inherited from class java.lang.Object**

`clone, equals, finalize, getClass, hashCode, notify, notifyAll, toString, wait, wait, wait`

**Constructor Detail**

### TextLabelFactory

```
public TextLabelFactory()
```

:   Constructs a TextLabelFactory.


**Method Detail**

### setDebug

```
public void setDebug(boolean debug)
```

:   Setting debug to true will turn on debugging (which prints data
    to standard out) for the GlyphSetFactory.

    :   **Parameters:**: `debug` - boolean value to turn debugging on or off.

---


### createLabel

```
public TextLabel createLabel(String text,
                             int width,
                             int height)
```

:   Uses Glyph objects to create a TextLabel with a Buffer containing
    an image based on the text String given as an argument.

    :   **Parameters:**: `text` - the string of characters to create an image for.: `width` - the width of the label in pixels.: `height` - the height of the label in pixels. **Returns:**: A TextLabel object with a Buffer holding the image.

---


### readGlyphSet

```
public void readGlyphSet()
                  throws GlyphException
```

:   Obtains a set of glyphs by using the default '.conf' configuration
    file in the fonts directory.

    :   **Throws:**: `GlyphException` - if a problem occurs while trying to read the set of glyphs from a file.

---


### readGlyphSet

```
public void readGlyphSet(String config)
                  throws GlyphException
```

:   Reads the config file given as an argument and use the info to
    create a GlyphSet.


---


|  |  |  |
| --- | --- | --- |
| **Overview** \| **Package** \| **Class** \| **Use** \| **Tree** \| **Deprecated** \| **Index** \| **Help** | |  |
| **PREV CLASS**   **NEXT CLASS** | **FRAMES**    **NO FRAMES**     **All Classes** |  |
| SUMMARY: NESTED \| FIELD \| CONSTR \| METHOD | DETAIL: FIELD \| CONSTR \| METHOD |  |


---

# *Copyright © 2007-2008*
